# Protecting visual short-term memory during maintenance: Attentional modulation of target and distractor representations

**DOI:** 10.1038/s41598-017-03995-0

**Published:** 2017-06-22

**Authors:** Marlies E. Vissers, Rasa Gulbinaite, Tijl van den Bos, Heleen A. Slagter

**Affiliations:** 1 University of Amsterdam, Department of Psychology, Department of Brain and Cognition, Amsterdam, Netherlands; 2 Centre National de la Recherche Scientifique, UMR 5549, Faculté de Médecine Purpan, Toulouse, France; 3 Université de Toulouse, Centre National de la Recherche Scientifique, UMR 5549, Faculté de Médecine Purpan, Toulouse, France; 4 Amsterdam Brain and Cognition center, University of Amsterdam, Amsterdam, Netherlands

## Abstract

In the presence of distraction, attentional filtering is a key predictor of efficient information storage in visual short-term memory (VSTM). Yet, the role of attention in distractor filtering, and the extent to which attentional filtering continues to protect information during post-perceptual stages of VSTM, remains largely unknown. In the current study, we investigated the role of spatial attention in distractor filtering during VSTM encoding and maintenance. Participants performed a change detection task with varying distractor load. Attentional deployment to target and distractor locations was tracked continuously by means of Steady-State Visual Evoked Potentials (SSVEPs). Analyses revealed that attention strongly modulated the amplitude of the second harmonic SSVEP response, with larger amplitudes at target compared to distractor locations. These attentional modulations commenced during encoding, and remained present during maintenance. Furthermore, the amount of attention paid to distractor locations was directly related to behavioral distractor costs: Individuals who paid more attention to target compared to distractor locations during VSTM maintenance generally suffered less from the presence of distractors. Together, these findings support an important role of spatial attention in distractor filtering at multiple stages of VSTM, and highlight the usefulness of SSVEPs in continuously tracking attention to multiple locations during VSTM.

## Introduction

Goal-directed behavior relies heavily on the ability to temporarily store goal-relevant information in visual short-term memory (VSTM). It is well known that attention plays a critical role in selective storage of goal-relevant information in VSTM, by preventing distractors from occupying the limited-capacity VSTM storage space^1–6^. Selective filtering of goal-irrelevant information benefits VSTM performance by increasing the likelihood that goal-relevant information is accurately maintained in VSTM^7^. While fronto-parietal attentional networks and the basal ganglia play an important role in gating access to VSTM^6, 8, 9^, it is less clear whether attentional filtering during VSTM also relies on local modulations of activity in sensory regions^10^. Relatedly, it is still unclear to what extent attention also helps to protect VSTM content during postperceptual stages of VSTM, such as maintenance. In the present study, we addressed these questions and examined the role of attention in processing of relevant and irrelevant visuospatial information during both VSTM encoding and maintenance.

Recent studies show that attentional selection of goal relevant information does not only involve enhancement of goal-relevant sensory processing, but also entails concurrent suppression of goal-irrelevant sensory processing^11–13^. Interestingly, reliance on these attentional mechanisms has been shown to vary across individuals as a function of working memory capacity (WMC). While high working memory capacity (WMC) individuals simultaneously enhance sensory processing of relevant information and suppress processing of distracting information, low WMC individuals exhibit impaired distractor suppression, and primarily enhance processing of relevant sensory information^13, 14^. Studies on attentional selection in the context of VSTM have reported a similar pattern: high VSTM capacity individuals are generally more efficient at suppressing distracting information compared to low VSTM capacity individuals^1, 6^. While current evidence for the role of attentional filtering during VSTM encoding is based on attentional modulation of task-irrelevant information^15^, enhancement of processing of relevant information during VSTM has received less attention. This renders it unclear if successful VSTM performance selectively relies on suppression of irrelevant sensory processing, or may also involve enhancement of goal-relevant sensory processing^11–13, 16, 17^.

A second outstanding question is whether attention continues to play a role in protecting the contents of VSTM after encoding, i.e., during VSTM maintenance, and if so, whether similar attentional mechanisms are at play during encoding and maintenance of information in VSTM. Existing research on the involvement of spatial attention during postperceptual stages of VSTM typically used retrocues during the VSTM delay period^18–23^, or presented novel distractors throughout the delay interval after encoding^24, 25^. Yet, retrocues typically provide an instruction to perform additional attentional manipulations on stored representations during maintenance^18, 21^. Hereby, retrocues thus introduce additional attentional modulations that are not necessarily inherent to VSTM, and may interfere with ongoing maintenance of information in VSTM. Therefore, the effect of retrocues on performance and neural activity during VSTM maintenance may not necessarily generalize to regular VSTM undisturbed by an attentional cue. Although retro-cueing studies have revealed large effects of further attentional manipulations of stored representations on VSTM performance, it remains unclear to what extent attention also plays a role during typical VSTM in the absence of attentional cues or novel distractors presented during VSTM delay.

Another line of research suggesting that attention also plays a role during postperceptual stages of VSTM is based on the sensory recruitment hypothesis, which posits that attention and VSTM rely on similar neural representations that eventually serve to control motor behavior^26–29^. A related hypothesis postulates that attention and VSTM share priority maps^30–35^. Priority maps are modulated through selective weighting of task-(ir)relevant features represented as topographic locations in space. These modulations remain present until a particular cognitive or behavioral goal is achieved^36^, implying that they should persist during post-perceptual stages of VSTM^31, 32^. In support of this hypothesis, several studies demonstrated attentional enhancement of sensory processing at stored locations during maintenance^15, 37–42^, for example during attentional refreshing o﻿f information stored in VSTM^43–45^. Furthermore, fMRI studies have revealed the presence of task-relevant representations in sensory cortices during VSTM maintenance^46, 47^, where the strength of sensory activation during retention was shown to predict the quality of a memory representation^48^. Yet, these findings were typically observed during maintenance of temporally segregated information^49^, or were measured for a single item at a time^15, 48^. Knowledge on sensory modulations during VSTM of more cluttered displays that also contain task-irrelevant distractors is presently lacking. This leaves unclear how sensory representations contribute to VSTM in the case of more complex spatial stimulus configurations as used in typical change-detection tasks, and that better represent crowded situations in everyday life. Thus, whereas priority maps have been proposed to optimize encoding and maintenance of goal-relevant information by acting as an attentional filter^31^, empirical evidence on their involvement in distractor suppression during VSTM is presently lacking.

**Figure 1:**
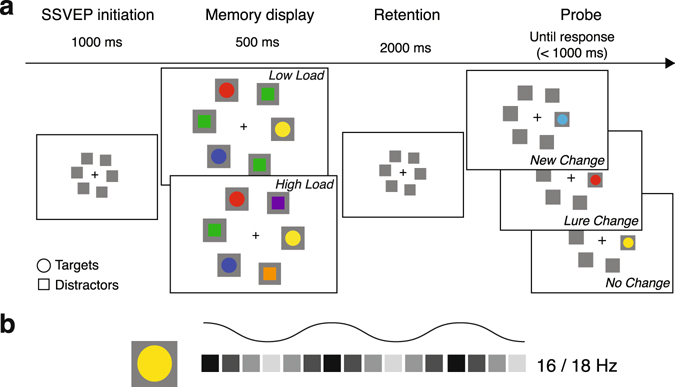
Experimental design. (**a**) Graphical display of the change-detection task with distractors. In this example targets were circles, and distractors were squares. In each trial, participants were shown a memory display with homogenously (low distractor load; top panel) or heterogeneously colored distractors (high distractor load; bottom panel). Distractor load was manipulated block-wise. After a retention interval, a probe stimulus was presented at one of the target locations. The probe could either have a new color (new change trials; top panel), a color of one of the target stimuli at a non-probed location (lure trials; middle panel), or a color identical to the target stimulus at that location (no change; bottom panel). (**b**) Stimulus contrast of the placeholders at stimulus locations was manipulated in a sinusoidal fashion from black to white to elicit SSVEPs at 16 and 18 Hz. Target and distractor locations flickered at different frequencies.

In the current study, we aimed to shed more light on the role of attentional dynamics in distractor filtering during VSTM encoding and maintenance. Given that attention modulates sensory activity in a retinotopically specific fashion^31, 32^, we focused on the role of spatial attention in distractor filtering during VSTM. Importantly, we assessed the role of attention during VSTM without possible confounds introduced by retrocues and measured ongoing attentional modulations of multiple, simultaneously presented sensory representations of target and distractor stimuli. This way, our approach yields important insight in the role of attention for VSTM in more realistic situations in which participants need to simultaneously encode and remember multiple relevant items that first need to be selected in the presence of goal-irrelevant information. Participants performed a change-detection task in which they were asked to remember the color of three target stimuli presented among distractors and indicate if the color of a subsequent probe stimulus matched the color of the target stimulus presented at that location (Figure 1a). To investigate distractor suppression during VSTM, we varied distractor load by manipulating distractor color similarity. In the ‘low distractor load’ condition, all distractors had the same color, whereas in the ‘high distractor load’ condition, each distractor had a unique color. On half of the trials, the color of the probe was different from the color of the target at the probed location (change trials). On half of the change trials, the color of the probe stimulus was identical to the color of a stimulus at a non-probed target location (‘lure trials’). Lure trials were introduced to encourage subjects to encode both the location and the color of each target stimulus.

Attentional allocation to relevant (target) and irrelevant (distractor) locations over time was measured via steady state visual evoked potentials (SSVEPs) that were elicited by placeholders at each stimulus location, as SSVEP amplitude has been shown to increase with attention^50^. Placeholders flickered during VSTM encoding and maintenance with unique frequency tags for relevant and irrelevant locations. Critically, this approach allowed us to continuously track attentional allocation across multiple relevant and irrelevant locations during both VSTM encoding and retention, without interfering with ongoing cognitive performance. We predicted the following patterns of results. First, at the behavioral level, we predicted that VSTM performance would be lower in the high vs. the low distractor load condition^51^, and we predicted that the effect of distractor load would be related to individual VSTM capacity^3^. Second, at the neural level, we predicted to find distractor suppression, reflected in reduced SSVEP responses at distractor compared to target locations during both VSTM encoding and maintenance, in particular on high compared to low distractor load trials. Third, we expected that the amount of attention paid to the distractors would have a direct effect on behavioral performance, such that individuals exhibiting stronger suppression of distractor representations during VSTM would show a reduced effect of distractor load on VSTM performance.

## Results

Forty-four participants completed the experiment. One participant was excluded because of chance-level performance on the change detection task, and another participant was excluded because of technical issues during EEG data collection. Three participants were excluded from further analyses because they made eye movements towards the flickering placeholders on more than 30% of the trials, and two were excluded because their EEG data was severely contaminated by blink and/or muscle artifacts. Our final sample for behavioral analysis consisted of 37 participants (M = 21.8 years; 26 F). The average partial symmetry span score on the complex working memory task was 30.5 across participants (SD 7.31; range 12–42). Two additional participants showed no SSVEP in the raw EEG data (power at the flicker frequencies was indistinguishable from power at neighboring frequencies; determined based on visual inspection of frequency spectrum of the sixteen most posterior channels; see the Methods section for details) and were excluded from further SSVEP analyses. Our final sample for SSVEP analyses thus consisted of 35 participants (M = 21.6 years; 26 F).

### The effect of distractor load on VSTM performance

**Figure 2:**
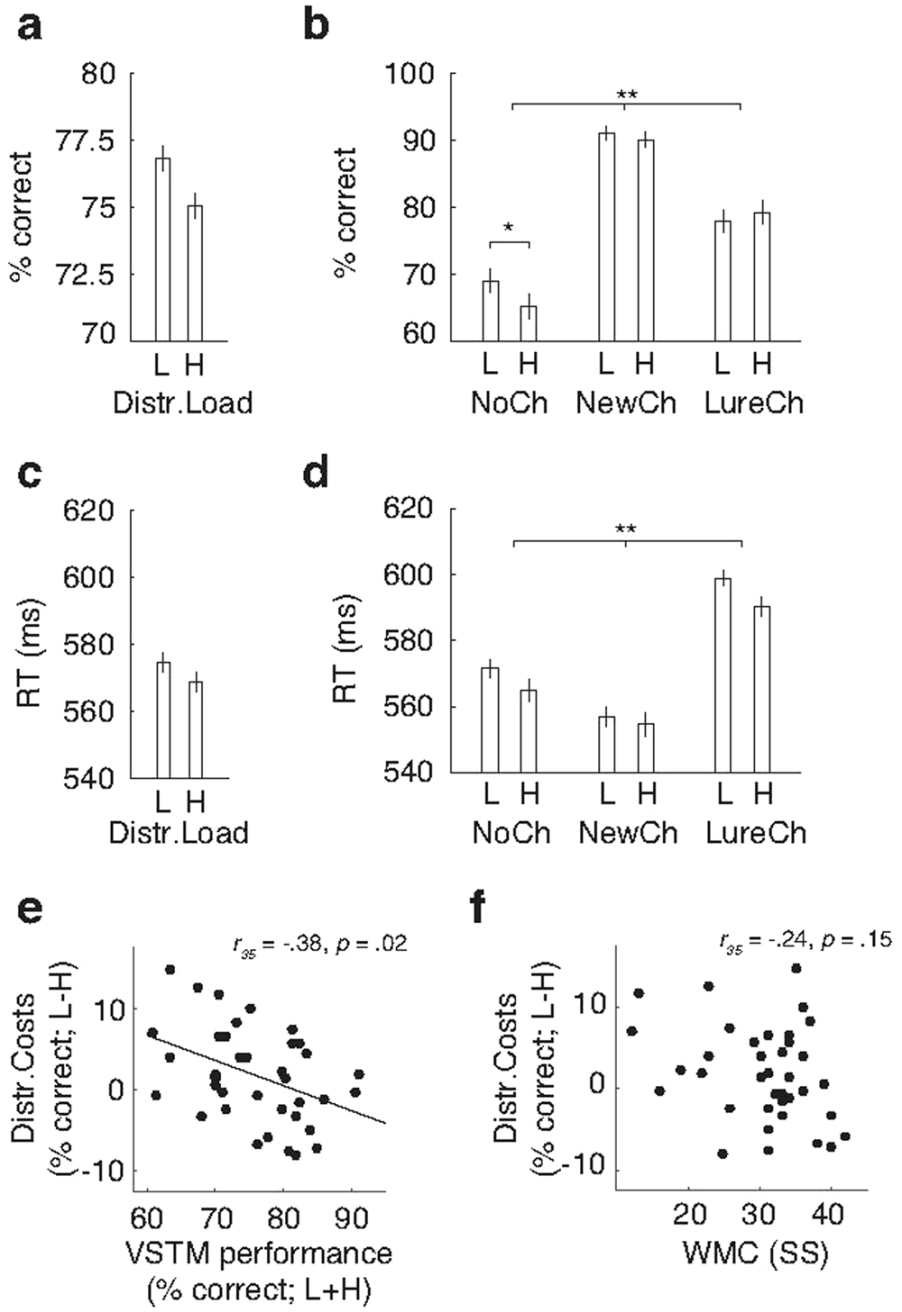
Behavioral performance on the change-detection task. (**a**) Accuracy of performance shown separately for low and high distractor load trials (averaged across trial type). (**b**) Accuracy of performance shown separately per trial type (no change; new change; lure change) and condition (low vs. high distractor load). Performance accuracy was lowest on no-change trials. Only in these trials, impaired performance was observed in high compared to low distractor load trials. (**c**) Response times on the task shown separately for low and high distractor load trials (averaged across trial type). (**d**) Response times per trial type (no change; new change; lure change) and condition (low vs. high distractor load). Response times differed across trial types, but were not affected by distractor load. (**e**) VSTM capacity (average performance on low and high distractor load trials) negatively predicted the effect of distractor load on VSTM performance (performance on high minus low distractor load trials), such that a high distractor load generally impaired performance more in low vs. high capacity individuals. (**f**) WMC as measured on the symmetry span task was not significantly related to the effect of distractors on performance. Error bars represent the standard error of the mean.

To examine our first prediction that VSTM performance would be lower in the high vs. the low distractor load condition, we conducted a repeated measures ANOVA with distractor load (low; high) and trial type (no change; new change; lure change) as within-subject factors. This analysis revealed no main effect of distractor load on accuracy of performance (F_1,36_ = 1.851, *p* = 0.182, see Figure 2a), but instead showed a significant interaction between distractor load and trial type (F_1.9,67.5_ = 3.868, *p* = 0.025). This interaction was due to a selective effect of distractor load on performance on no-change trials: Post-hoc comparisons revealed that accuracy on no-change trials was lower for the high (M = 65%) compared to low distractor load condition (M = 69%; t_36_ = −2.456, *p* = 0.02; see Figure 2b), whereas the effect of distractor load was not significant on new change and lure change trials (all *p*’s > 0.37). Notably, we also observed a main effect of trial type (F_1.5,54.1_ = 41.233, *p* < 0.001), reflecting much poorer performance on no-change (M = 67%) compared to new change (M = 91%; t_36_ = −9.081, *p* < 0.001) and lure change trials (M = 79%; t_36_ = −4.453, *p* < 0.001). Further, planned comparisons showed that performance was worse on lure (M = 79%) compared to new change trials (M = 91%; t_36_ = −4.627, *p* < 0.001). Analyses of participants’ sensitivity to detect a change under different distractor loads (low; high) across trial types (new change; lure change) using d’^52^ yielded similar results (main effect of trial type; F_1,36_ = 13.377, *p* < 0.001, with poorer performance on lure change trials). As perfect binding of color to location would have resulted in equal performance on new change and lure change trials, this indicates that participants did not always perfectly bind stimulus color to location. Thus, distractor load only affected performance accuracy in no-change trials, the trial type on which accuracy of performance was the lowest.

Reaction time analyses also revealed a main effect of trial type (F_1.9,68.6_ = 38.331, *p* < 0.001, see Figure 2d), but no main effect or interactions with distractor load (see Figure 2c). Post-hoc t-tests showed that participants were slower to respond on no-change compared to change trials (t_36_ = 2.747, *p* = 0.020), as well as lure change trials (t_36_ = −5.826, *p* < 0.001). Planned contrasts showed that participants were also slower on lure change compared to new change trials (t_36_ = −8.573, *p* < 0.001), indicative of imperfect binding of color to location. The effect of distractor load on performance was thus selective to accuracy of performance on the relatively difficult no-change trials.

### Individual differences in the effect of distractor load on accuracy of performance

Based on previous research on individual differences in the effect of distractors on performance as a function of VSTM capacity^1^, we also predicted that sensitivity to distraction would vary as a function of individual VSTM capacity. To test this prediction, we examined if VSTM capacity measured on the change detection task, and WMC as measured on a symmetry span task (administered in a separate session), predicted the effect of distractor load on accuracy of performance (distractor costs; % correct on low minus high distractor load trials). This analysis showed a negative correlation between VSTM capacity and behavioral distractor costs (r_35_ = −0.380, *p* = 0.020; Figure 2e), in line with our prediction and previous research showing that individuals with a high VSTM capacity are better at filtering out distractors^1, 15^ (repeating this analysis post-hoc including the no-change trials only yielded a similar result: r_35_ = −0.501, *p* = 0.002). As can also be seen in Figure 2e, low capacity individuals showed a stronger impairment in the high distractor load condition, whereas high capacity individuals showed poorer performance in the low distractor load condition (reflected in negative distractor costs). The correlation between the effect of distractor load on VSTM performance and WMC measured on the symmetry span task was in the same direction, albeit not significant (r_35_ = −0.242, *p* = 0.148; see Figure 2f; repeating this analysis post-hoc using only the no-change trials did reveal a significant relationship between WMC and distractor costs; r_35_ = −0.331, *p* = 0.047).

### SSVEP results: modulation by spatial attention

**Figure 3:**
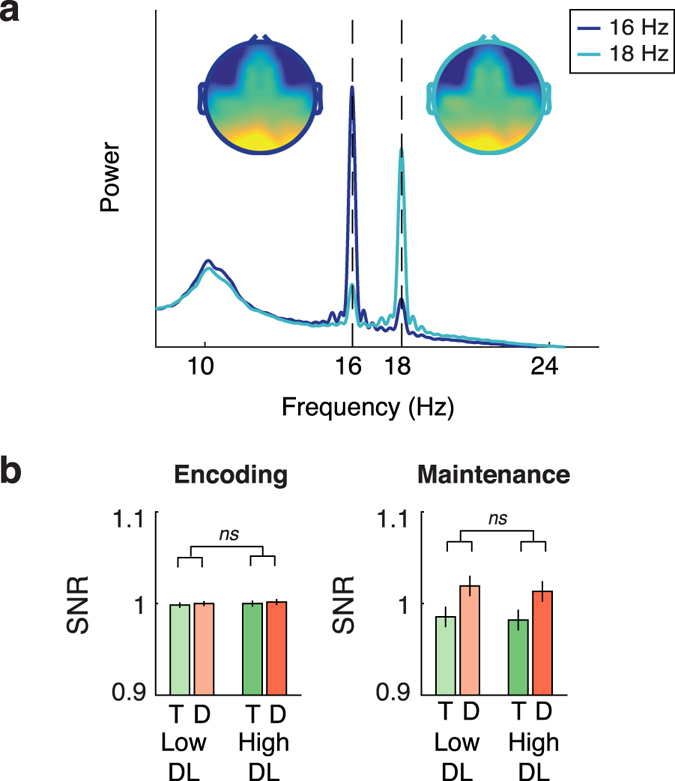
SSVEP responses at the fundamental frequency. (**a**) Frequency spectrum and topography of the RESS time series optimized for 16 (dark blue) and 18 Hz (light blue) activity, computed using the condition-average data in the time-window during which the flickering placeholders were on screen and the SSVEP was stable (−500 to 2500 ms; the y-axis reflects arbitrary power units as the RESS time series were computed based on multivariate source separation). At the group level, topographies show strong overlap between frequencies. The frequency spectra confirm distinct differences between the group average RESS time series for 16 and 18 Hz in terms of their frequency content. (**b**) SSVEP response (computed as the normalized SNR) for target (T; green) and distractor (D; red) locations, showing no significant differences in SSVEP power between target and distractor locations. Distractor load (DL; low vs. high; light vs. dark shades respectively) also did not affect SSVEP power. Error bars represent the standard error of the mean.

The interval- and condition-average frequency spectrum and topographical distribution of the SSVEPs at the flicker frequencies is displayed in Figure 3a. Attentional deployment to target vs. distractor locations under different distractor loads was assessed using a repeated measures ANOVA with location (target; distractor), distractor load (low; high) and interval (encoding; maintenance) as within-subject factors. In contrast to our second prediction, no difference in SSVEP response at the flicker frequencies was observed at target compared to distractor locations (F_1,34_ = 3.40, *p* = 0.074). If anything, SSVEP amplitude was numerically higher at distractor locations (see Figure 3b). Other main effects or interactions were also not significant (all *p*’s > 0.10). Thus, these results provide no support for modulation of attention to target and distractor locations during VSTM encoding or maintenance, or an effect of distractor load on the distribution of spatial attention.

**Figure 4:**
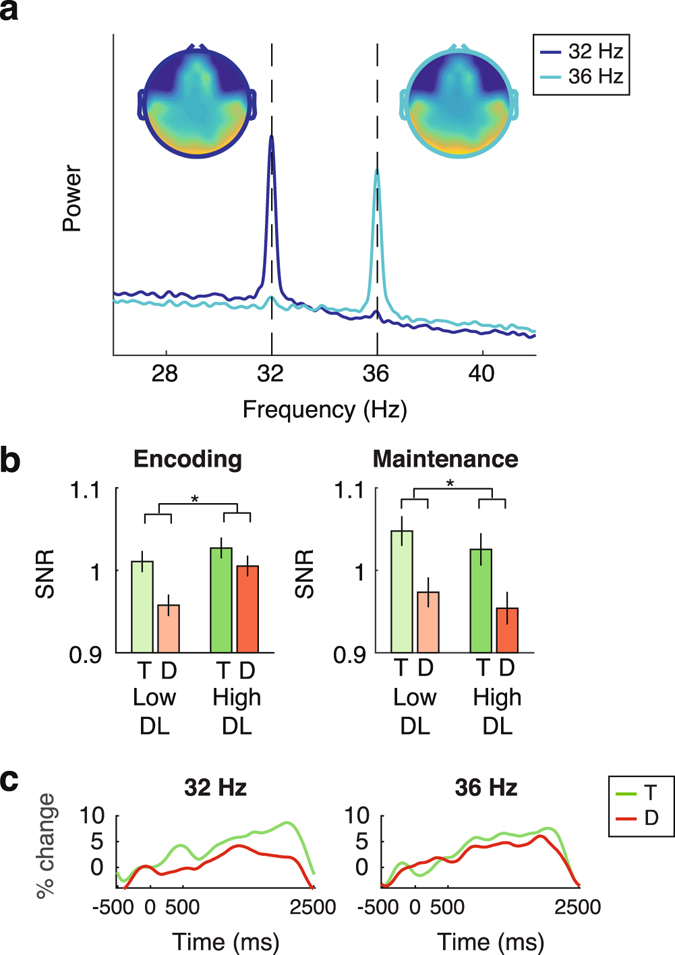
SSVEP responses at the second harmonic. (**a**) Frequency spectrum and topography of the RESS time series optimized for the second harmonics of the SSVEP at 32 (dark blue) and 36 (light blue) Hz, computed using the condition-average data in the time-window during which the flickering placeholders were on screen and the SSVEP was stable (−500 to 2500 ms), using the condition-average data (the y-axis reflects arbitrary power units as the RESS time series were computed based on multivariate source separation). The average topographies of the two frequencies show strong overlap, but differ from the topographies of the SSVEP at the fundamental frequency (see Figure 3a). The frequency spectra show distinct differences between the two RESS time series in terms of their frequency content. (**b**) SSVEP response (computed as the normalized SNR) for target (T; green) and distractor (D; red) locations. SSVEP responses were stronger at target compared to distractor locations during both VSTM encoding and maintenance. Distractor load (DL; low vs. high; light vs. dark shades respectively) did not affect SSVEP power. (**c**) Time course of the SSVEP, displayed as the % change in activity relative to the baseline (−200 to 0 ms), for the target (green) and distractor (red) locations at 16 Hz (left panel), and 18 Hz (right panel). The time course was obtained via the Hilbert transform of the bandpass filtered RESS time series (FWHM 3 Hz) for each frequency and condition (targets and distractor locations). Prior to baseline normalization, for each time point, the bandpass-filtered and Hilbertized power at the neighboring frequencies (spaced ± 2, 2.5, 3 Hz distance) was subtracted from the power at the frequencies of interest (similar to the computation of the SSVEP response shown in **b**). Error bars represent the standard error of the mean.

Given that previous research has shown that attentional modulations of SSVEP amplitude may be restricted to the second harmonic of the SSVEP^53^, we next examined the presence of attentional modulations at target and distractors locations using the second harmonic of the SSVEP. The interval- and condition-average frequency spectrum and topography of the second harmonic of the SSVEP are displayed in Figure 4a. A repeated measures ANOVA revealed a significant main effect of location (F_1,35_ = 7.318, p = 0.011). Follow-up comparisons revealed an increased SSVEP response at target compared to distractor locations during encoding (t_34_ = 2.224, *p* = 0.040), as well as maintenance (t_34_ = 3.320, *p* = 0.011; see Figure 4b). No other main effects or interactions were significant, suggesting that at the group level, distractor load did not differentially affect attentional allocation to target or distractor locations during VSTM encoding and/or maintenance. In short, spatial attention continuously modulated target and distractor representations during VSTM independently of distractor load, and this was selectively captured in the second harmonic of the SSVEPs.

### Individual differences in distractor processing during VSTM

In line with previous findings^1, 54^ and our first prediction, analysis of the behavioral data showed that individual short-term memory capacity was significantly related to sensitivity to distractors. We therefore also assessed whether the attentional modulations observed in the second harmonic of the SSVEP were related to individual differences in the effect of distractor load on behavior. To this end, we included distractor costs as a covariate in the repeated measures ANOVA in which we tested the effect of location (target; distractor) and distractor load (low; high) on amplitude of the second harmonic of the SSVEP (note that we collapsed across time intervals since these showed no effect in the ANOVA, see previous section). Inclusion of behavioral distractor costs revealed a significant three-way interaction between location, condition, and distractor costs (F_1,33_ = 5.384, *p* = 0.027), suggesting that the way in which distractor load affected attentional deployment to target vs. distractor locations predicted behavioral distractor costs.

**Figure 5:**
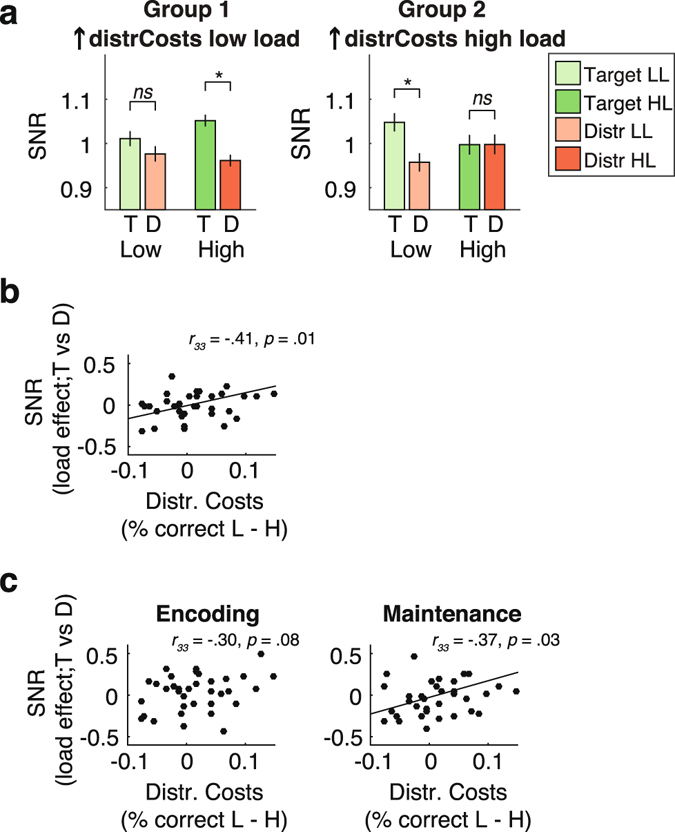
Individual differences in attentional modulation of the second harmonic SSVEP relate to VSTM performance. (**a**) Graphical display of the three-way interaction between the effect of location (T vs. D), condition (low vs. high), and behavioral distractor costs, by means of a median split of participants based on distractor costs. Group 1 consists of participants with low distractor costs (group-average distractor costs were negative in this subgroup), who performed better in the high compared to the low distractor load condition. Participants in group 2 showed high (positive) distractor costs, and thus performed better in the low compared to high distractor load condition. Within each subgroup, differential allocation of attention across target and distractor locations was apparent in the condition in which participants performed best, but was not observable in the condition in which they failed to prevent distractors from interfering with VTSM performance. A post-hoc significant negative correlation between behavioral distractor costs and the difference in attentional allocation to target vs. distractor locations in the high vs. low distractor load condition (double subtraction) confirmed the interaction observed in the ANOVA, (**b**) averaged across VSTM encoding and maintenance, and (**c**) separately for the encoding and maintenance interval. Individuals who paid more attention to target vs. distractor locations under high distractor loads generally displayed reduced VTSM impairments in the high compared to the low distractor load condition. Error bars represent the standard error of the mean.

To visualize the observed interaction, we defined two groups of participants based on a median split of distractor costs: Individuals who, on average, showed larger distractor costs in the low distractor load condition (group 1), and individuals who, on average, showed larger distractors costs in the high distractor load condition (group 2; see Figure 5a). This median split illustrates that modulations of spatial attention were only visible in the distractor load condition in which participants performed best, but were not apparent in the condition in which performance was more severely impaired by distractors. This was confirmed by a post-hoc correlation test in which we correlated the behavioral effect of distractor load to the effect of distractor load on the difference in attentional allocation to target vs. distractor locations (r_33_ = −0.41; *p* = 0.01; see Figure 5b). Although our analysis did not reveal a significant interaction with the effect of time (encoding vs. maintenance), we post-hoc assessed whether the relationship between the effect of distractor load on differential modulation of target and distractor locations during VSTM was present during encoding and maintenance alone, as this could strengthen support for the notion that attention filtering during VSTM maintenance contributes to performance. Results showed that the cross-subject relationship between the effect of distractor load on VSTM performance and on attention to target and distractor locations, was at trend level during VSTM encoding (r = −0.30, *p* = 0.08), and reached significance during VSTM maintenance (r = −0.37, *p* = 0.03; see Figure 5c). This suggests that differential attentional deployment across target and distractor locations helps to protect the stored contents of VSTM during VSTM maintenance.

To determine if the observed relationship between the effect of distractor load on performance and attentional allocation to target vs. distractor locations was driven by changes in attention to target locations, changes in attention to distractor locations, or both, we post hoc separately correlated the difference in the SSVEP amplitude in the high vs. low load condition at target (or distractor) locations with the effect of distractors on behavior. This revealed no evidence for a selective relationship between the effect of distractor load on target amplitude or distractor amplitudes (all *p*’s < 0.25), suggesting that individual differences in how distractor load affected performance did not selectively reflect individual differences in distractor suppression or target enhancement^13^. Instead, individual differences in the effect of distractor load on the *relative* distribution of attention to target versus distractor locations predicted the effect of distractors on individual performance.

## Discussion

Previous research has shown that attention plays a critical role in encoding goal-relevant information in VSTM by preventing interference from distracting information. In the current study, we examined the distribution of spatial attention (as measured by SSVEP amplitude) across task-relevant and task-irrelevant locations during both encoding and maintenance stages of VSTM under conditions of high and low distractor load. We report three main findings. First, in line with previous research^5, 15^, we found that individual differences in the effect of distractor load on behavior were related to VSTM capacity, such that low-capacity individuals suffered more when distractor load was high compared to high-capacity individuals. Second, we found that attention strongly modulated the amplitude of the second harmonic SSVEP response, with larger amplitudes at target compared to distractor locations during VSTM maintenance. Lastly, cross-subject analyses revealed a relationship between the deployment of spatial attention to target vs. distractor locations and the effect of distractor load on behavioral performance. Specifically, individuals who showed stronger differentiation of attention to target versus distractor locations, showed less distractor-related performance impairments. Together, these findings indicate that spatial attention not only determines the quality of sensory representations during VSTM encoding, but may also help to preserve sensory representations during VSTM maintenance.

Extending previous studies demonstrating the importance of attention during VSTM encoding in the presence of distraction^1, 4, 5, 15^, we observed differential attentional deployment across target and distractor locations that commenced during VSTM encoding, and importantly, persisted during VSTM maintenance. This finding may suggest that selective attention not only supports VSTM during encoding, but also plays an important role in preserving VSTM contents during VSTM maintenance. Moreover, it corroborates previous findings showing that attention can filter irrelevant perceptual information by modulating sensory activity^4, 7, 14^, and reveals that such sensory modulations persist during post-perceptual stages of VSTM, i.e., during maintenance of stored information. This may suggest that retention of information and attending to information rely on shared neural populations or mechanisms in sensory regions^26, 29, 31, 33^, in line with the sensory recruitment account of VSTM^10^.

Thus far, the role of attention during VSTM maintenance has mostly been examined by presenting distractors during the delay interval, i.e. after target encoding^24, 25, 55, 56^, or by presenting retrocues or instructions to refresh certain stored representations during the maintenance interval^7, 21, 44, 57^. Our findings indicate the involvement of attentional modulations during typical maintenance of information in VSTM regardless of the need to suppress new perceptual input during the VSTM delay interval. This is important as findings from a recent behavioral study revealed that filtering of distractors presented along goal-relevant information during VSTM encoding is unrelated to filtering of newly presented distractors during the VSTM delay period^58^, suggesting these processes may rely on different neural mechanisms. Furthermore, our results reveal that attention plays an essential role in accurate maintenance of representations in VSTM, even when there is no need for further attentional selection of stored representations during maintenance (e.g., following retrocues^21, 59^). By probing attentional modulations of target and distractor locations throughout VSTM without introducing novel task-irrelevant distractors or retrocues during the delay interval, we were able to demonstrate that attentional deployment also supports typical VSTM maintenance. Future research is necessary to determine whether attentional filtering of distraction during the encoding interval and attentional filtering of distraction presented during maintenance indeed rely on different neural mechanisms.

Another main finding of the current study was that the extent to which an individual allocated attention to target relative to distractor locations during VSTM predicted the extent to which distracting information impaired VSTM performance. Specifically, we found that individuals who paid more attention to target versus distractor locations generally showed less distractor-related interference of VSTM performance than individuals who did not. These results may suggest that attentional modulations of sensory activity form a powerful mechanism that helps to preserve information in VSTM even when distraction is no longer physically present. Taking the differential allocation of spatial attention across target and distractor locations to reflect an attentional filter^31^, this finding extends previous observations that individual differences in distractor filtering during encoding predict distractor-related impairments in VSTM performance^1, 6^.

In line with our findings, a recent ERP study reported impaired filtering abilities in low-capacity individuals, that were, importantly, accompanied by larger sensory responses to probe stimuli at distractor locations immediately following VSTM encoding in low-capacity, relative to high-capacity individuals^15^. Based on these results, the authors suggested that the impaired filtering ability in low-capacity individuals arises from the inability to recover from momentary attentional capture by task-irrelevant distractors^15, 60^. Our findings suggest that distracting information may continue to affect attentional allocation to target vs. distractor locations long after this information has disappeared, throughout VSTM maintenance, and that individual differences in ongoing, not just momentary, attentional allocation to relevant and irrelevant locations predict filtering ability during VSTM.

Importantly, across individuals, relative attentional deployment to target vs. distractor locations predicted the effect of distractors on VSTM performance, but attention to target or distractor locations alone did not. Although this result corroborates previous findings showing the close interplay between relative enhancement vs. suppression of goal-(ir)relevant information during attentional selection^12^, we did not find evidence for a selective failure to suppress distraction in low-capacity individuals^13, 15^. Thus, relative differences between target enhancement and distractor suppression may be a better predictor of VSTM performance accuracy than suppression of distractors alone.

Notably, distractor load exerted different effects on VSTM performance in low- and high-capacity individuals. At the group level, we observed the expected pattern of increased distractor-related performance impairments for high compared to low distractor loads, although this effect was selective to no-change trials^61^. However, the effects of distractor load on VSTM performance varied in direction in a systematic fashion based on VSTM capacity: Low-capacity individuals performed worse in the high compared to low distractor load condition, whereas the opposite was true for high-capacity individuals, who performed best under high distractor loads. It is conceivable that low- and high-capacity individuals may have used different strategies to filter distraction, resulting in different effects of distractor load on performance^62^. Yet, our observation that optimal task performance was accompanied by increased attention to target vs. distractor locations across all participants, speaks against this scenario. An alternative explanation could be that the effect of memory load and distractor load interacted. This would be in line with the proposal that the ability to focus attention improves under task conditions of high perceptual load, but deteriorates under conditions of high cognitive load (e.g., when working memory is loaded)^63^. According to this account, when cognitive load is low (e.g., for high VSTM capacity individuals), a high perceptual load (high distractor load condition) leads to more successful filtering. When cognitive load is high on the other hand (e.g., for low-capacity individuals), this results in an overall impairment of filtering ability, leading to poorer performance under high compared to low distraction conditions. In other words, when taking VSTM capacity as an inverse proxy of cognitive load (low-capacity individuals will have experienced a higher cognitive load), and distractor load as the perceptual load induced by the task, our pattern of findings is in line with the idea that cognitive load and perceptual load interact to affect the ability to filter distractors^63, 64^.

An important, but unanticipated finding was that the observed attentional modulations were selective to the second harmonic of the SSVEP response. We had also expected to find effects of attention on the fundamental frequency component of the SSVEP^14, 50^. It is possible that we did not observe effects of our attentional manipulation on the fundamental SSVEP response because the SSVEPs were generated by flickering placeholders around the stimulus locations (see Figure 1a) instead of flickering stimuli presented at the stimulus locations themselves. This may have resulted in surround suppression of the placeholder (surrounding the stimulus location), thereby also suppressing the fundamental frequency response of the SSVEP^65, 66^. Given that surround suppression was likely stronger for attended compared to irrelevant locations^66^, this could also explain why we observed a trend for a reduced (rather than enhanced) fundamental SSVEP response at target compared to distractor locations (see Figure 2b).

Yet, we are not the first to report the selective presence of attentional modulations in the second harmonic instead of the fundamental frequency of the SSVEP^53, 67, 68^. An alternative explanation for why the second harmonic SSVEP response was more sensitive to attention in our and other studies is that fundamental and harmonic SSVEP responses may be generated by different neural populations and/or brain regions that may respond differently to particular stimulus properties and/or attentional manipulations. For instance, the fundamental frequency response may be primarily generated in striate cortex, whereas the harmonics of the SSVEP may be generated in extrastriate regions or more dorsal and anterior regions^53, 69^. The different topographical and functional characteristics of the fundamental and harmonic frequency components of the SSVEP have been speculated to reside in differential involvement of subcortical (fundamental) versus cortico-cortical (harmonic) communication^69^, or in increased top-down control over generators of the harmonic compared to the fundamental SSVEP component^53, 69^. Yet, actual knowledge on the neuroanatomical basis of the fundamental and harmonic frequency components of the SSVEP is presently remarkably limited^53, 69, 70^. Future research is necessary to determine the influence of various bottom-up and top-down effects on the fundamental frequency and second harmonic of the SSVEP and to increase understanding of the functional differences between these responses^53^, for instance through systematic comparisons of the effect of bottom-up (e.g., surround-suppression^71^) and top-down factors (e.g., attention) on the same frequency SSVEP as a fundamental or harmonic response to a flickering stimulus^69^. This will not only help to form more accurate hypotheses in experiment design, but will also provide important insights into properties of the visual system related to bottom-up and top-down modulations of visual processing.

The use of SSVEPs in the current study enabled us to continuously track attention to target and distractor locations during VSTM. Yet, the current design does not permit investigation of the temporal dynamics of attentional allocation to each individual target and distractor location. Contradictory to the conception of a multifocal and tonically divided attentional focus^50^, recent studies indicate that attention may rhythmically sample across locations and objects^72, 73^. Potentially, our finding of prolonged and continuous differential attentional deployment to target and distractor locations during VSTM is a result of averaged rhythmic modulations of SSVEP amplitude across locations and trials^74^. As we tagged all target and all distractor locations with the same frequency on every trial, we were not able to disentangle attentional allocation to individual stimulus locations using the present task design. The temporal dynamics of the deployment of attention during VSTM is an interesting and important venue for future research. Moreover, previous studies on attentional filtering in service of VSTM have indicated an important role for frontal regions and the basal ganglia in preventing distractors from interfering with VSTM^6, 9^. An interesting question for future research concerns the involvement of these regions in modulating sensory representations to filter distracting information during VSTM retention. Lastly, additional studies are necessary to further examine the functional relevance of the distractor-related attentional modulations observed in the current study^36^. Based on the observed correlation between distractor costs and attentional deployment during VSTM, we cannot infer whether the allocation of attention is functional and serves storage of information in VSTM^39, 75^, or alternatively, whether attentional allocation is driven by VSTM contents but is not necessary for accurate performance^76, 77^. Studies in which the behavioral consequences of disrupting retinotopic modulation of spatial attention during VSTM maintenance are investigated^78^, will be informative in this respect.

To summarize, in this study, we show distractor-related modulations of spatial attention during VSTM that last during VSTM maintenance and predict effects of distraction on VSTM performance. Attention may thus help to protect the contents of VSTM during post-perceptual stages of VSTM.

## Methods

### Participants

Forty-four participants were recruited using the online participant recruitment system of the Psychology Department of the University of Amsterdam and participated in return for monetary compensation or course credits. Participants were neuropsychologically healthy (assessed based on self-report), and were screened for color-blindness, right-handedness, epilepsy and migraine. Informed consent was obtained from all participants before the start of the experiment. The procedure for the experiment was approved by the local ethics committee of the University of Amsterdam, and was in accordance with the approved guidelines and regulations. As detailed in the results section, our final sample consisted of 37 participants (M = 21.8 years; 26 F).

### Experimental task

Participants performed a VSTM change detection task (partially modeled after^79^) in which they had to remember the color and location of three targets that were presented in the presence of three distractors (see Figure 1a). The total number of stimuli (six) exceeded average VSTM capacity^54^, necessitating filtering of distractors for accurate storage of the targets^15^. Stimulus presentation and response registration were controlled using Psychtoolbox^80^ and Matlab (The MathWorks). Stimuli were displayed on a 24-inch monitor with a 144 Hz refresh rate. Target and distractor stimuli had different shapes (squares/circles; 0.9/1.02 degrees visual angle (dva) diameter), and the assignment of shape (squares/circles) to stimulus type (targets/distractors) was counterbalanced across participants. All stimuli had clearly distinct, but equiluminant colors (difference between colors < 10 cd/m2). Stimuli were presented on square placeholders (width/height 1.2 dva), positioned on a ring around fixation (placed at 4 dva from fixation). Placement of stimuli across locations occurred in a pseudorandom fashion such that target stimuli were always placed across both hemifields. Participants thus had no predictive information regarding the location of the targets and distractors preceding presentation of the memory display, and needed to reactively select the targets from distractors based on stimulus shape. Distractor load was manipulated through distractor color similarity. In the ‘low distractor load’ condition, all distractors had the same color, whereas in the ‘high distractor load’ condition, each distractor had a unique color (see Figure 1a).

In order to track attentional allocation across space and time, we used frequency tagging of stimulus locations to evoke SSVEPs separately for target and distractor locations^14^. Hereto, stimulus placeholders changed luminance from black to white in a sinusoidal fashion (see Figure 1b). The flicker frequency assigned to each placeholder in a given trial was based on the stimulus type presented at the placeholder (target vs. distractor), such that target and distractor locations were each tagged with a unique flicker frequency. The tagging frequencies used were spaced closely together in the frequency spectrum (16 and 18 Hz) to minimize perceptual differences between the placeholders that could affect attentional deployment, and were outside the frequency range of endogenous oscillatory activity usually observed during VSTM, such as theta and alpha activity^81–83^. The assignment of frequencies to target vs. distractor locations was counterbalanced across conditions (low vs. high distractor load). Prior to the start of the experiment, participants received instructions about the shape of targets and distractors (the assignment of stimulus type to shape was constant throughout the experiment for each participant), and were explicitly informed that distractor colors were irrelevant for accurate performance on the task. Participants received no information about the distractor manipulation between conditions.

Each trial started with a 1000 ms presentation of six flickering placeholders in order to obtain a stable SSVEP response to the flickering placeholders. Subsequently, a memory array with distractors was presented within the placeholders for 500 ms, which was followed by maintenance interval of 2000 ms during which the stimuli were removed from the screen but the flickering placeholders remained present. Placeholders flickered throughout the entire trial until presentation of the probe. The probe stimulus was presented on a placeholder at a randomly selected target position, and its color was different from the color of the probed target in the memory display on half of the trials (change trials).

To ensure that participants bound stimulus colors to stimulus locations, we manipulated the color of the probe stimulus such that on half of the change trials the probe color was identical to the color of a stimulus at a non-probed target location (‘lure trials’). On the other half of change trials, the probe would adopt a ‘new’ color (‘new change trials’; see Figure 1a). Binding of stimulus color to location would result in an equal performance on lure and new change trials, whereas a lack of color-location binding (e.g., if participants would simply verbalize target colors) should result in impaired performance on lure trials. This manipulation thus enabled the assessment of the degree to which participants bound stimulus color to location during VSTM. Lure and new change trials were randomly interspersed among no-change trials.

On presentation of the probe, participants indicated whether the color of the probe was identical to the color of the target at the probed location by pressing one of two response buttons placed on the right armrest of the chair (right index finger for no change; right middle finger for change). After a response was made or when the response interval (1000 ms) elapsed, the placeholders were removed from the screen. An inter trial interval (ITI) of 1000 ms preceded the next trial. A central fixation-cross was shown during the entire trial. Participants were instructed to keep their eyes at fixation at all times.

The experiment consisted of 6 blocks of 64 trials. Distractor load was manipulated block-wise, and the condition (low vs. high distractor load) with which the experiment started was counterbalanced across participants. Participants were allowed a self-paced break after every 32 trials, during which their average reaction times and accuracy level were presented on the screen. Participants were reminded to respond as accurate and fast as possible, and were motivated to respond faster if their average accuracy levels exceeded 90% to prevent ceiling effects in accuracy of performance.

Throughout the entire experiment, eye movements were monitored using a Tobii eye tracker (Tobii AB, Stockholm, Sweden). Gaze data were analyzed online, so that the experimenter could remind the participant to keep fixation in case the participant repeatedly broke fixation during trials.

### Procedure

Participants were seated at a 90 cm distance from the computer monitor. Before the start of the experimental task, four minutes of resting state data were recorded. During the resting state EEG recording, participants were asked to alternately watch a central fixation dot and close their eyes for one minute. Onset times for eyes closed and fixation were cued using an auditory cue. This sequence was repeated twice. Analyses of the resting state data were not included in the current report.

After completion of the resting state recording, participants were given extensive task instructions for the change detection task and performed a practice session to become acquainted with the task. The practice session consisted of four practice blocks of 16 trials each. In the first two practice blocks, immediate feedback was given on participants’ performance (’correct’,’incorrect’, or’too late’). In the last two practice blocks, there was no trial-wise feedback anymore, similar to the experimental task. Following the practice session, participants continued with the change detection task. Together, the practice and experimental EEG session lasted approximately one hour. At the end of the EEG session, participants were invited for a separate short behavioral session in which we administered a symmetry span task^84^ to obtain an independent index of participants’ complex visual working memory capacity^85^.

### EEG recording and preprocessing

EEG data were acquired at a sampling rate of 512 Hz using a Biosemi set-up with 64 channels, placed according to the 10–20 system. External reference electrodes were placed on the earlobes, to be used for off-line referencing of the data. External electrodes placed above and below the left eye were used to measure vertical eye movements and blinks (vertical electro-oculogram; VEOG). Electrodes placed at the outer canthi were used to measure horizontal eye movements (horizontal electro-oculogram; HEOG).

Off-line, the EEG data were high-pass filtered at 0.5 Hz, and epoched from −2200 to 5000 ms around stimulus onset. The epoched data was manually inspected and trials with muscle artifacts, as well as blinks during presentation of the flickering placeholders (potentially impeding an SSVEP response) were excluded from further analysis. Gaze data were used to detect trials containing eye-movements during the encoding and maintenance interval of the task (deviations of ≥1.5° visual angle away from fixation for 50 consecutive ms or longer). The cleaned datasets for each participant contained 313 trials (SD 43.5 trials) on average (81.4% of the total number of trials in the task; SD 11.3%).

### SSVEP analyses: spatiotemporal filtering

The SSVEP and ongoing spontaneous oscillatory activity usually have overlapping frequency content, impeding separation of the SSVEP based on spectral characteristics of the data alone. In order to optimally extract SSVEPs, we exploited the distinct temporal (frequency) as well as spatial (scalp distribution) characteristics of the SSVEP signal with respect to the ongoing background activity. This was done through rhythmic entrainment source separation (RESS^86^). RESS belongs to a family of denoising source separation techniques that can be optimized to extract specific spatiotemporal features of the data^87^. RESS comprises the computation of a spatial filter that effectively maximizes the explained variance for a specified feature of the data, in our case the frequency-specific SSVEP signal, relative to a reference signal, in our case broadband ongoing electrophysiological activity. RESS was performed on the concatenated epochs spanning the time window during which the flickering stimuli were presented on the screen (excluding the first 500 ms during which the SSVEP was still building up, resulting in a window of −500 to 2500 ms around stimulus presentation. Power at the SSVEP frequency was isolated by band-pass filtering the concatenated trials using a narrow-band filter centered at the SSVEP frequency of interest (frequency-domain Gaussian filter kernel; FWHM = 0.5 Hz). The broadband EEG data was taken as the reference data. Time-averaged covariance matrices were computed separately for the SSVEP data at the center frequency (S) and the reference data (R). Generalized eigenvalue decomposition of the matrix product R^−1^S was used to construct frequency-specific spatial filters (RESS components). The eigenvector (i.e., channel weights) with the largest eigenvalue was selected as the spatiotemporal filter at the tagging frequency. This eigenvector (column vector with values representing channel weights) was multiplied by the original channel time series to obtain a component time series. As can be seen in Figure 3a, the frequency content of the resulting component time series is highly specific to the tagging frequencies used.

Given that topographies of SSVEPs may differ across participants and frequencies^14^, we computed the spatial filters separately for each participant and flicker frequency. Furthermore, as activity elicited at different stimulus positions may project differently onto the scalp^88^, the procedure described above was performed separately for the 14 unique stimulus configurations used in the experiment by using subsets of trials. This finally resulted in two frequency-specific component time courses for every trial optimized for each flicker frequency and trial-specific stimulus configuration. Note that construction of the spatial filters was done on the condition-average data (collapsed across target vs. distractor locations and distractor load), and was thus independent of potential effects of experimental manipulations.

As the main potential danger of RESS is overfitting^86^, resulting in time series reflecting noise instead of SSVEPs, we only included participants for whom inspection of the frequency spectrum of the raw data showed a peak at the SSVEP frequencies on at least one of the 16 most posterior channels. Furthermore, we excluded the most anterior channels prior to RESS to prevent contamination of the component time series by frontal electromyographic (EMG) artifacts at the frequency of interest. RESS was performed using the 43 remaining channels (see Figure 3a). The effect of experimental manipulations on SSVEP amplitude was assessed by comparing SSVEP amplitude across experimental conditions (targets vs. distractors; low vs. high distractor load) and time intervals (encoding 0–500 ms; maintenance 500–2500 ms). Hereto, we performed an FFT on the data and computed the trial-average frequency spectrum for every condition (using a frequency resolution of 0.1 Hz). In order to avoid an effect of power-law scaling of EEG data on our results, we expressed the power at each flicker frequency as signal-to-noise-ratio (SNR), which was computed as the power at the SSVEP peak relative to the power at neighboring frequencies (averaging the power at frequencies spaced ± 0.4, 0.5 and 0.6 Hz distance from the SSVEP frequency of interest^88^). Given that previous research has shown that attentional modulations of SSVEP amplitude may be restricted to the second harmonic of the SSVEP^53^, we also examined the presence of attentional modulations of stimulus locations on the second harmonic of the SSVEP (32 and 36 Hz). Hereto, separate spatial filters were constructed and resultant component time series were analyzed as described before. The SNR at 32 and 36 Hz was computed using the average power at neighboring frequencies spaced ± 2, 2.5, and 3 Hz distance from the SSVEP frequency (neighboring frequencies for the harmonics were spaced at a larger distance from the SSVEP frequency to take into account the wider frequency response of the component time series; see Figure 4a vs. 3a). For both the fundamental and harmonic component of the SSVEP, we normalized the condition-specific SNR according to the average SNR for each interval and frequency^89^. In the following, whenever we mention SSVEP response, we refer to the normalized SNR of the SSVEP computed as outlined here.

### Statistical analyses

Our first prediction was that VSTM performance would be lower on high compared to low distractor load trials. To test this prediction, we examined the effects of our manipulation of distractor load (low vs. high) and probe type (no change; new change; lure change) on accuracy (% correct) and speed (RT in ms) of performance with separate repeated measured ANOVAs. In case of significant effects, follow-up paired t-tests were used to assess the statistical significance of the contrasts of interest. Planned comparisons were used to assess the difference in VSTM performance on lure and new change trials in order to assess color-location binding. All behavioral analyses were computed on the cleaned behavioral data after exclusion of trials containing eye movements, uninformed responses (RTs < 150 ms), and response omissions. Working memory capacity (WMC) was measured as the partial symmetry span score on the symmetry span task^90^. We expected that effects of distractor load on performance would differ between high- and low-capacity individuals. Therefore, we correlated the effect of distractor load on performance accuracy (distractor costs; decline in % correct for low vs. high distractor loads) with VSTM capacity (measured as average VSTM performance across distractor loads) and WMC (measured on the symmetry span task).

In order to investigate attentional deployment to target and distractor locations during VSTM, we normalized SSVEP amplitudes within each frequency (see previous section) and subsequently collapsed across tagging frequencies^89^. Statistical analyses were performed on the correct and cleaned trials only. To determine the effects of attention, we subjected the condition specific normalized SSVEP response (at the fundamental frequency or second harmonic) to a repeated measures ANOVA with the factors location (target; distractor), condition (low; high distractor load), and interval (encoding 0–500 ms; maintenance 500–2500 ms). In case of significant effects, post-hoc t-tests were conducted to test the difference between different levels of the factor of interest. Lastly, as we expected individual differences in the mechanisms involved in attentional filtering^13, 14^, we examined whether attentional modulation of target and distractor locations was related to the effect of distractors on behavior by including behavioral distractor costs as a covariate in our analysis. An alpha level of 0.05 was used as the significance criterion for all statistical analyses.

### Data availability statement

The datasets generated during and/or analyzed during the current study are stored in the University of Amsterdam repository, and are available from the corresponding author on reasonable request.
